# Supplementary material for: Modeling glioblastoma heterogeneity as a dynamic network of cell states
Source: Mol Syst Biol. 2021 Sep 16;17(9):e10105. doi: 10.15252/msb.202010105 (PMC8444284; doi:10.15252/msb.202010105)
Supplement: Supplementary file 6 — Source Data for Figure 5 [file MSB-17-e10105-s004.zip › Figure5A_sourcedata/GSEA_3071/hallmarks_stateV.GseaPreranked.1623742546329/HALLMARK_ADIPOGENESIS.html]

Details for gene set HALLMARK\_ADIPOGENESIS[GSEA]

|  || Dataset | state43071 |
| Phenotype | NoPhenotypeAvailable |
| Upregulated in class | na\_neg |
| GeneSet | HALLMARK\_ADIPOGENESIS |
| Enrichment Score (ES) | -0.39454135 |
| Normalized Enrichment Score (NES) | -2.115685 |
| Nominal p-value | 0.0 |
| FDR q-value | 5.010856E-4 |
| FWER p-Value | 0.004 |
Table: GSEA Results Summary

  

Fig 1: Enrichment plot: HALLMARK\_ADIPOGENESIS      
 Profile of the Running ES Score & Positions of GeneSet Members on the Rank Ordered List

  

| PROBE | GENE SYMBOL | GENE\_TITLE | RANK IN GENE LIST | RANK METRIC SCORE | RUNNING ES | CORE ENRICHMENT || 1 | ITGA7 |  |  | 37 | 1.644 | 0.0269 | No |
| 2 | CDKN2C |  |  | 223 | 1.143 | 0.0151 | No |
| 3 | APLP2 |  |  | 396 | 0.952 | 0.0018 | No |
| 4 | GPX4 |  |  | 701 | 0.746 | -0.0410 | No |
| 5 | SORBS1 |  |  | 763 | 0.717 | -0.0379 | No |
| 6 | ELOVL6 |  |  | 925 | 0.649 | -0.0553 | No |
| 7 | HADH |  |  | 1242 | 0.548 | -0.1046 | No |
| 8 | TST |  |  | 1321 | 0.528 | -0.1086 | No |
| 9 | ACADM |  |  | 1475 | 0.487 | -0.1278 | No |
| 10 | AGPAT3 |  |  | 1556 | 0.469 | -0.1335 | No |
| 11 | GPHN |  |  | 1721 | 0.433 | -0.1559 | No |
| 12 | UCP2 |  |  | 1779 | 0.422 | -0.1581 | No |
| 13 | CCNG2 |  |  | 1780 | 0.422 | -0.1494 | No |
| 14 | VEGFB |  |  | 1781 | 0.422 | -0.1407 | No |
| 15 | SLC25A10 |  |  | 1783 | 0.422 | -0.1322 | No |
| 16 | SCARB1 |  |  | 1920 | 0.401 | -0.1499 | No |
| 17 | ADCY6 |  |  | 2018 | 0.385 | -0.1606 | No |
| 18 | ATL2 |  |  | 2098 | 0.374 | -0.1680 | No |
| 19 | BCL6 |  |  | 2130 | 0.370 | -0.1663 | No |
| 20 | SLC27A1 |  |  | 2193 | 0.362 | -0.1707 | No |
| 21 | GPD2 |  |  | 2222 | 0.359 | -0.1686 | No |
| 22 | PFKL |  |  | 2241 | 0.358 | -0.1646 | No |
| 23 | DGAT1 |  |  | 2278 | 0.353 | -0.1642 | No |
| 24 | ADIPOR2 |  |  | 2360 | 0.341 | -0.1727 | No |
| 25 | TOB1 |  |  | 2442 | 0.330 | -0.1814 | No |
| 26 | CRAT |  |  | 2458 | 0.327 | -0.1776 | No |
| 27 | UCK1 |  |  | 2563 | 0.315 | -0.1910 | No |
| 28 | RTN3 |  |  | 2671 | 0.302 | -0.2052 | No |
| 29 | NKIRAS1 |  |  | 2773 | 0.292 | -0.2185 | No |
| 30 | ACO2 |  |  | 2807 | 0.288 | -0.2189 | No |
| 31 | COX8A |  |  | 2998 | 0.270 | -0.2498 | No |
| 32 | PPM1B |  |  | 3007 | 0.268 | -0.2457 | No |
| 33 | NDUFAB1 |  |  | 3018 | 0.267 | -0.2421 | No |
| 34 | RMDN3 |  |  | 3042 | 0.265 | -0.2411 | No |
| 35 | ATP1B3 |  |  | 3158 | 0.254 | -0.2579 | No |
| 36 | MRPL15 |  |  | 3203 | -0.252 | -0.2611 | No |
| 37 | PEMT |  |  | 3209 | -0.253 | -0.2568 | No |
| 38 | STOM |  |  | 3230 | -0.256 | -0.2554 | No |
| 39 | SNCG |  |  | 3480 | -0.296 | -0.2970 | No |
| 40 | COQ5 |  |  | 3556 | -0.308 | -0.3050 | No |
| 41 | DNAJB9 |  |  | 3721 | -0.334 | -0.3295 | No |
| 42 | NDUFA5 |  |  | 3784 | -0.346 | -0.3342 | No |
| 43 | RIOK3 |  |  | 3855 | -0.359 | -0.3402 | No |
| 44 | GHITM |  |  | 3970 | -0.382 | -0.3541 | No |
| 45 | LIFR |  |  | 4158 | -0.426 | -0.3811 | No |
| 46 | APOE |  |  | 4229 | -0.449 | -0.3853 | Yes |
| 47 | IFNGR1 |  |  | 4240 | -0.452 | -0.3778 | Yes |
| 48 | POR |  |  | 4261 | -0.460 | -0.3722 | Yes |
| 49 | TANK |  |  | 4272 | -0.466 | -0.3645 | Yes |
| 50 | MDH2 |  |  | 4294 | -0.471 | -0.3587 | Yes |
| 51 | SDHB |  |  | 4321 | -0.477 | -0.3539 | Yes |
| 52 | ABCA1 |  |  | 4333 | -0.483 | -0.3460 | Yes |
| 53 | GADD45A |  |  | 4399 | -0.500 | -0.3481 | Yes |
| 54 | OMD |  |  | 4412 | -0.505 | -0.3400 | Yes |
| 55 | GRPEL1 |  |  | 4446 | -0.519 | -0.3356 | Yes |
| 56 | PDCD4 |  |  | 4460 | -0.524 | -0.3272 | Yes |
| 57 | MGST3 |  |  | 4461 | -0.524 | -0.3164 | Yes |
| 58 | SCP2 |  |  | 4581 | -0.577 | -0.3273 | Yes |
| 59 | ARL4A |  |  | 4593 | -0.582 | -0.3174 | Yes |
| 60 | CAVIN2 |  |  | 4602 | -0.590 | -0.3067 | Yes |
| 61 | DHRS7 |  |  | 4615 | -0.601 | -0.2966 | Yes |
| 62 | REEP5 |  |  | 4692 | -0.642 | -0.2979 | Yes |
| 63 | ANGPT1 |  |  | 4825 | -0.735 | -0.3080 | Yes |
| 64 | CMBL |  |  | 4847 | -0.753 | -0.2964 | Yes |
| 65 | ENPP2 |  |  | 4876 | -0.784 | -0.2856 | Yes |
| 66 | DNAJC15 |  |  | 4887 | -0.795 | -0.2711 | Yes |
| 67 | ITIH5 |  |  | 4934 | -0.850 | -0.2623 | Yes |
| 68 | ME1 |  |  | 4982 | -0.899 | -0.2528 | Yes |
| 69 | RAB34 |  |  | 5085 | -1.115 | -0.2492 | Yes |
| 70 | CAVIN1 |  |  | 5116 | -1.195 | -0.2303 | Yes |
| 71 | SLC1A5 |  |  | 5145 | -1.294 | -0.2089 | Yes |
| 72 | CHCHD10 |  |  | 5160 | -1.361 | -0.1835 | Yes |
| 73 | COL15A1 |  |  | 5190 | -1.490 | -0.1582 | Yes |
| 74 | NABP1 |  |  | 5203 | -1.558 | -0.1283 | Yes |
| 75 | MGLL |  |  | 5249 | -2.003 | -0.0955 | Yes |
| 76 | COL4A1 |  |  | 5266 | -2.279 | -0.0515 | Yes |
| 77 | SSPN |  |  | 5284 | -2.752 | 0.0021 | Yes |
Table: GSEA details [plain text format]

  

Fig 2: HALLMARK\_ADIPOGENESIS: Random ES distribution      
 Gene set null distribution of ES for **HALLMARK\_ADIPOGENESIS**
